# The role of spectrophotometry in the diagnosis of melanoma

**DOI:** 10.1186/1471-5945-10-5

**Published:** 2010-08-13

**Authors:** Paolo A Ascierto, Marco Palla, Fabrizio Ayala, Ileana De Michele, Corrado Caracò, Antonio Daponte, Ester Simeone, Stefano Mori, Maurizio Del Giudice, Rocco A Satriano, Antonio Vozza, Giuseppe Palmieri, Nicola Mozzillo

**Affiliations:** 1 National Cancer Institute, Naples, Italy; 2 Unit of Dermatology, Second University of Naples, Italy; 3 Institute of Biomolecular Chemistry-CNR, Sassari, Italy

## Abstract

**Background:**

Spectrophotometry (SPT) could represent a promising technique for the diagnosis of cutaneous melanoma (CM) at earlier stages of the disease. Starting from our experience, we further assessed the role of SPT in CM early detection.

**Methods:**

During a health campaign for malignant melanoma at National Cancer Institute of Naples, we identified a subset of 54 lesions to be addressed to surgical excision and histological examination. Before surgery, all patients were investigated by clinical and epiluminescence microscopy (ELM) screenings; selected lesions underwent spectrophotometer analysis. For SPT, we used a video spectrophotometer imaging system (Spectroshade^® ^MHT S.p.A., Verona, Italy).

**Results:**

Among the 54 patients harbouring cutaneous pigmented lesions, we performed comparison between results from the SPT screening and the histological diagnoses as well as evaluation of both sensitivity and specificity in detecting CM using either SPT or conventional approaches. For all pigmented lesions, agreement between histology and SPT classification was 57.4%. The sensitivity and specificity of SPT in detecting melanoma were 66.6% and 76.2%, respectively.

**Conclusions:**

Although SPT is still considered as a valuable diagnostic tool for CM, its low accuracy, sensitivity, and specificity represent the main hamper for the introduction of such a methodology in clinical practice. Dermoscopy remains the best diagnostic tool for the preoperative diagnosis of pigmented skin lesions.

## Background

The incidence of melanoma in Europe has been steadily increasing, more rapidly than for any other cancer, during recent decades. Incidence of melanoma deeply varies across Europe, with the highest incidence in Scandinavian countries.

Lifetime risk of developing melanoma within the entire caucasian population is estimated to be 1:535 individuals. Incidence is higher in individuals with fair skin who have been exposed to high levels of UV-B radiation. Mortality due to metastatic melanoma has increased rapidly in both males and females; such a disease accounts for only 4% of skin cancers, but for 79% of skin-cancer related deaths. In particular, mortality rates are higher in males than in females; mortality has doubled in males over the past 25 years. Again, death rates are higher in individuals with fair skin. From 2002 to 2006, the median age at death due to metastatic melanoma of the skin was 68 years. Both incidence and mortality rates are highest in Australia and New Zealand. The 5-year survival rate for patients with advanced melanoma is less than 10% [1-6].

In melanoma patients, survival is strongly related to tumor thickness; earlier diagnosis and complete excision of lesions may thus lead to a decline in mortality for such a disease. The most effective tool in fighting malignant melanoma is detecting the disease with a depth of ≤ 1 mm (Breslow thickness) and without ulceration, which is associated with an excellent prognosis (95% 5-year survival rate), in comparison to detection of a malignant melanoma with a depth of > 4 mm (40% 5-year survival rate), as reported by the American Joint Committee on Cancer [7].

Diagnostic accuracy for pigmented skin lesions using the naked eye has been estimated to be about 60% [8]. To improve the accuracy of melanoma diagnosis, a variety of diagnostic instruments has been developed: dermoscopy, multispectral imaging, confocal laser microscopy, ultrasonography, optical coherence tomography, or magnetic resonance imaging [9].

From its introduction in 1990s, dermoscopy or epiluminescence microscopy (ELM), a non-invasive tool for cutaneous screenings, has become the most popular technique for early diagnosis of melanoma [10-33], also reducing the excess of cases addressed to excision biopsy [34]. However, ELM is an useful tool in expert hands. As stated in a systematic review by Kittler and colleagues, dermoscopy does improve the diagnostic accuracy for melanoma in comparison with eye-based inspection, when used by experienced examiners only [35]. Recentely, a meta-analysis evaluating diagnostic accuracy for melanoma confirmed that sensitivity was much higher for dermoscopy (0.90; 95% confidence interval [CI], 0.80-0.95) than for naked eye examination alone (0.71; 95% CI, 0.59-0.82), with an estimated difference of about 0.18 (95% CI, 0.09-0.27; P = 0.002) [36]. Conversely, there was no statistical evidence of a significant difference in specificity [dermoscopy 0.90 (95% CI, 0.57-0.98) *versus *naked eye examination 0.81 (95% CI 0.48-0.95); difference 0.09 (95% CI, 0.06-0.23, P = 0.18)] [36]. In addition, a meta-analysis of 30 studies using dermoscopy showed a sensitivity of 0.88 (95% CI, 0.87-0.89) and a specificity of 0.86 (95% CI, 0.85-0.86) [37].

Since sensitivity and specificity of dermoscopy significantly vary among different studies without reaching the 100% of validity, additional non-invasive techniques, such as spectrophotometric intracutaneous analysis (SIA) and spectrophotometry (SPT), have been introduced to improve early diagnosis of melanoma.

The SIA technique (SIAscopy, as named by Moncrieff et al. [38]), which was developed using the model of light transport [39,40], is aimed at differentiating melanomas from non-melanoma skin cancers, benign melanocytic naevi and other pigmented lesions such as seborrhoeic keratosis [38].

The spectrophotometry is a method that evaluate the color of a lesion by measuring its reflectance as a function of the wavelength. A pioneer in this field was Marshall [41] who described a comprehensive analysis of pigmented lesions under both ultraviolet and infrared radiations. In such a study, he stated that the infrared photographs tends to discriminate the different types of lesions, with melanoma showing a relatively high degree of correlation with low infrared reflectance.

The SPT (Spectroshade, MHT Verona, Italy) system allows to extract information regarding the skin microarchitecture, like the distribution, position, and quantity of blood, collagen and melanin throughout the epidermidis and papillary dermis, converting data from reflected near infrared radiation into *in vivo *histological images of the lesion [38]. The SPT system provides information including a series of 15 multispectral images into the near infrared bandwidth. On this regard, permanence of the image into the infrared area of the spectrum has been demonstrated to represent a significant indication of malignancy of the pigmented lesion under examination. Seven parameters (mean reflectans, MR; variegation, V; area, A; dark area ratio, DAR; dark island reflectance, DA; dark distribution factor, DDF) are processed automatically by software, describing the pigmented lesion features. Three spectral areas play a major role in quantification of all these parameters: 584 nm, where the highest contrast between lesion and the surrounding skin is determined; 650-750 nm, where a pigmented lesion can be better discriminated since the light adsorption due to melanine is much higher than that due to blood; 750-950 nm, where the lesion color is quantified in the near infrared, a spectral area invisible to the clinician's eye. After exposing the skin to visible light and infrared radiation, the spectrophotometric system converts the reflected radiations into *in vivo *histological images of the lesion by means of a sophisticated computer algorithm. Such an image allow to extract information regarding the microarchitecture of the cutaneous alteration under examination.

Starting from our experience in the use of both ELM and SPT, we intend to define the role of spectophotometric diagnosis in the early detection of cutaneous melanoma.

## Methods

In recent years, physicians of the Melanoma Cooperative Group at the National Cancer Institute of Naples have performed a considerable amount of visits for cutaneous screening (about 10.000 visits per year). For the evaluation of clinically-relevant pigmented lesions, epiluminescence microscopy (ELM) was carried out using a Molemax II (Derma Instruments, Vienna) video-dermatoscope.

According to the criteria provided by our group for the treatment of cutaneous pigmented lesions [42], each patient was interviewed in order to obtain a personal profile (date of birthday, place of residence, type of work, history of sun exposure and sun burning, familial recurrence of skin lesions), before undergoing a clinical total body skin examination. Cutaneous pigmented lesions were classified according to the macroscopic ABCDE criteria; when at least two of the ABCDE criteria occurred, individuals were addressed to ELM-based dermoscopy, in order to generate a risk classification. Such an evaluation was made by expert dermatologists (at least 3 years of experience). Lesions were classified as non-melanocytic lesions (such as angiokeratoma, verrucous naevus, pigmented basal cell carcinoma, seborrhoeic keratosis, angioma, keratoacantoma, solar keratosis, etc.) or melanocytic lesions [such as compound naevus, intradermal naevus, papillomatous compound naevus, Spitz naevus, blue naevus (without pigment network); junctional naevus, lentigo simplex, pigmented spindle cell naevus of Reed, naevus spilus, cockarde naevus, atypical naevus, malignant melanoma (with pigment network)] (Table 1).

**Table 1:** ELM-based classification of cutaneous lesions and corresponding histology

CUTANEOUS LESIONS	HISTOLOGICAL LESIONS
**Non-melanocytic lesions**	angiokeratoma, verrucous nevus, pigmented basal cell carcinoma, seborrhoeic keratosis, angioma, keratoacantoma, solar keratosis, etc.

**Melanocytic lesions** **(without pigment network)**	compound nevus, intradermal nevus, papillomatous compound nevus, Spitz nevus, blue nevus.
**(with pigment network)**	junctional nevus, lentigo simplex, pigmented spindle cell nevus (Reed nevus), nevus spilus, cockarde nevus, atipycal nevus, **melanoma**.

**Other lesions**	squamous carcinoma, trichoepithelioma, Becker's nevus, Ota's nevus, Ito's nevus, Halo nevus.

Melanocytic lesions were further classified as very low, low, medium, high, and very high risk lesions [18]. Characterization and classification of each lesion were based on the presence or absence of typical ELM features (Table 2) [18]. According to our working formulation [42], each category was further classified as A (not clinically suspicious) or B (clinical suspicious), on the basis of the clinical history of the lesion (a suspicious history is defined when a lesion presents a modification of its clinical parameters during the past 6 months).

**Table 2:** ELM-based risk classification for cutaneous pigmented lesions

MELANOCYTIC LESIONS	ELM FEATURES
**Type 1** **(very high risk)**	Lesion with a pigment network and any of the classical ELM features specific for melanoma (pseudopods, radial streaming, blue-gray veil, atypical vessel, etc.)

**Type 2** **(high risk)**	Lesion with a pigment network and subtle new ELM features that may suggest melanoma but often are also seen in atypical nevi.

**Type 3** **(medium risk)**	Lesion with a pigment network carrying subtle perturbations that can be detected in atypical naevus as well as in melanocytic hyperplasia.

**Type 4** **(low risk)**	Lesion with a benign appearing network.

**Type 5** **(very low risk)**	Lesion with a benign appearing network and with a globular pattern or another benign ELM pattern.

Surgical excision was advised for high and very high risk lesions. In type A of medium, low, and very low risk lesions, surgery may be justified on the basis of cosmetic and/or functional reasons (in absence of contraindications for surgical excisions). Anyway, patients with medium risk lesions entered a close follow-up program. Surgery was always recommended for all type B lesions [42]. In case of advise for surgery of a melanocytic lesion, patients were invited to participate in our study; a written informed consent was obtained before patient's enrollment into the study.

Fifty-four individuals with cutaneous pigmented lesions, who were addressed to surgery after ELM classification, were also evaluated by spectrophotometry (SPT), using the SpectroShade system (MHT, Verona, Italy). Comparisons between SPT and ELM results were conducted in order to verify whether SPT may highlight, through multispectral image analysis, new features of pigmented lesions (content and distribution of the adsorbing components of the skin, such as melanin and haemoglobin), which might be useful to improve the early diagnosis of melanoma. Table 3 summarizes the characteristics of the SPT classes and presents the correlation with the ELM risk classification.

**Table 3:** SPT diagnostic classes and corresponding ELM risk classes

SPT CLASSES	PROBABILITY OF MELANOMA	CORRESPONDING ELM RISK CLASS
**1**	no melanoma	Non Melanocytic and Type 5
**2**	doubtful melanoma (< 25%)	Type 4
**3**	suspected melanoma (< 75%)	Types 3 and 2
**4**	probable melanoma (> 75%)	Type 1

The study was reviewed and approved by the Ethycal Committee at the National Cancer Institute of Naples (Italy).

### Statistical methods

SPT sensitivity and specificity as well as positive and negative predictive values were determined according to a computer-assisted statistical analysis using the SPSS software for Windows, 8.0 version (SPSS Inc., Chicago, USA). Taking histology as standard, a lesion was considered true positive (TP) or true negative (TN) if results from SPT or ELM and histology were consistent (in other words, a concordance was achieved using such two diagnostic approaches). Conversely, a lesion was considered as false positive (FP) or false negative (FN) if histology did not confirmed the classification of SPT/ELM (in other words, SPT/ELM was positive but histology was negative or SPT/ELM was negative but histology was positive). Sensitivity was calculated as TP/TP+FN; specificity was calculated as TN/TN+FP; positive predictive value was calculated as TP/TP+FP; negative predictive value was calculated as TN/TN+FN.

## Results

In a period of one year, total-body skin examinations for detection of early malignant melanoma were carried out at National Cancer Institute of Naples; they allowed to identify cutaneous pigmented lesions to be addressed to surgical excision and histological examination in 54 patients [35 females and 19 males; median age, 41 (range, 19-73 years)]. Before surgery, lesions were evaluated by both ELM and SPT approaches.

According to ELM criteria [42], all 54 cutaneous pigmented lesions were classified as melanocytic lesions; according to the risk criteria (Table 3), they were classified as very low risk (N = 11; 20%), low risk (7; 13%), medium risk (1; 2%), high risk (21; 39%), and very high risk (14; 26%) lesions. The comparison between ELM-based classifications and histological results among the 54 excised lesions gave an agreement of 100% for very low risk lesions (6 papillomatosus compound naevus and 5 intradermal naevus), 100% for low risk lesions (7 junctional naevus or compound naevus), 100% for medium risk lesion (1 lentigo simplex), 90.4% for high risk lesions (19 atypical naevus and 2 Reed naevus), and 85.7% for very high risk lesions (12 melanoma and 2 atypical naevus). Overall, agreement between histology and ELM diagnosis was 92.5% (Table 4).

**Table 4:** Agreement between ELM diagnosis and histology

Type of lesion	ELM Diagnosis	Histological Diagnosis Positive/Negative	Agreement %
Non-melanocytic lesions	-	-/-	-
Melanocytic lesions	54	50/4	92.5
Very low risk	11	11/-	100
Low risk	7	7/-	100
Medium risk	1	1/-	100
High risk	21	19/2	90.4
Very high risk	14	12/2	85.7
TOTAL	54	50/4	92.5

Before surgery, the same 54 selected pigmented lesions were evaluated by SpectroShade software, which automatically classified them in four different classes: group 1, non melanoma; group 2, doubtful melanoma (< 25%); group 3, suspected melanoma (< 75%); group 4, probable melanoma (> 75%). Since criteria for classification into such groups appeared to be not particularly accurate, we decided to modify such a classification and correlate it to the ELM-based risk assessment of each specific cutaneous lesion. Therefore, we defined the following classes: group 1, non melanocytic and very low risk lesions; group 2, low risk lesions; group 3, medium and high risk lesions; group 4, very high risk lesions (Table 3).

Again, the comparison between SPT classifications and histological results among the 54 excised lesions gave an agreement of 66.6% (3 lesions) in group 1, 100% (5 lesions) in group 2, 57.1% (28 lesions) in group 3, and 44.4% (18 lesions) in group 4 (Table 5). Among all pigmented lesions, agreement between histology and SPT diagnosis was low (57.4%) (Table 5). Consistently, the agreement between ELM and SPT classifications was also quite low (overall, 68.5%) (Table 6).

**Table 5:** Agreement between SPT diagnosis and histology

Groups	SPT Diagnosis	Histological Diagnosis Positive/Negative	Agreement %
Group 1	3	2/1	66.6
Group 2	5	5/0	100
Group 3	28	16/12	57.1
Group 4	18	8/10	44.4

TOTAL	54	31/23	57.4

**Table 6:** Agreement between SPT and ELM classifications

Groups	SPT Diagnosis	ELM Diagnosis Positive/Negative	Agreement %
Group 1	3	2/1	66.6
Group 2	5	5/0	100
Group 3	28	16/12	57.1
Group 4	18	14/4	77.7

TOTAL	54	37/17	68.5

As summarized in Table 7, SPT-based classifications and histological results were evaluated for both sensitivity and specificity of this method among the 54 excised lesions. The sensitivity and specificity of SPT in detecting melanoma (group 4) were 66.6% and 76.2%, respectively; moreover, the positive and negative predict values were 44.4% and 88.9%, respectively (Table 7). Since the rate of SPT diagnoses was progressively higher as the thickness of the lesions increased (Table 8), the sensitivity and specificity of this method in detecting high risk lesions (atypical naevi and melanomas) remained lower than expected.

**Table 7:** Sensitivity and specificity for SPT in group 4 lesions

	Histology positive	Histology negative	
SPT positive	8	10	Sensitivity = 66.6
SPT negative	4	32	Specificity = 76.2

**Table 8:** Breslow thickness of melanomas diagnosed in the series of 54 cutaneous lesions

BRESLOW THICKNESS CATEGORIES	N. OF CASES	MEDIAN BRESLOW THICKNESS	RANGE
**< 1 mm**	**5**	**0.7**	**0.3-1.0**
	*1*	*1.0*	*-*
**1.01 to 2 mm**	**5**	**1.45**	**1.2-2.0**
	*5*	*1.45*	*1.2-2.0*
**2.01 to 4.0 mm**	**2**	**2.25**	**2.1-2.4**
	*2*	*2.25*	*2.1-2.4*
**> 4.01 mm**	**0**	**0**	**0**

**TOTAL**	**12**	**1.3**	**0.3-2.4**

The 8 cases diagnosed by SPT are indicated in *italic*.

## Discussion

Classification of pigmented lesions is actually based on both clinical evaluation, by naked-eye, and dermoscopy of the skin features; however, diagnosis strongly depends on the experience of physicians. At present, the ELM methodology, even in the hands of experienced examiners, does not have 100% sensitivity and specificity (overall, 88% sensitivity and 86% specificity [37]). To improve the early detection of melanoma, new diagnostic tools providing an automated classification of pigmented skin lesions (in other words, new devices which could be also used by non-expert physicians) have been proposed. Among others, the SPT is considered as the most promising method for such a purpose.

Although the series of analyzed patients in the present study was quite small, our findings seem to indicate that the SPT may have a limited role into the early diagnosis of melanoma (66.6% sensitivity; 76.2% specificity; 44.4% positive predicitive value; 88.9% negative predictive value). About sensitivity, one melanoma (0.8 mm breslow thickness, with a large dermal regression) was classified in group 1 (very low risk) by SPT; the other three melanomas (median breslow thickness, 0,4 mm; range 0,3-0,7 mm) were classified in group 2 by SPT. Similarly, specificity was quite low since only 8 out of 18 lesions, which have been classified in group 4 by SPT, were really malignant after histological diagnosis (consequently, the agreement with histology was also low).

Another important issue is the difficulty to perform the SPT evaluation on palpable lesions such as papillomatous naevi. In fact, 6 of these latter lesions and 3 dermal naevi were diagnosed in group 3 by SPT; therefore, this contributed to lower the agreement with the histology also in such a group.

Considering previous studies using the SPT approach, some difference was observed among the image acquisition systems of the various instruments. However, the Spectroshade^® ^(MHT, Verona, Italy), which was used for screening in our series, and the SIAscope (Astron Clinica, Cambridge, UK), which has been commonly used worldwide, are quite similar; both spectrophotometers are able to acquire images at wavelengths between 483-950 nm (Spectroshade) [43] or 400-1000 (SIAscope) [38] and both of them use computer algorithms to analyze quite similar parameters of the pigmented lesions. Therefore, it is possible to compare our results with those reported in literature, using these two spectrophotometric systems. Table 9 summarizes the results from the main studies published in past years [38,44-50]_._. Farina et al. (2000) [45] reported the first large study (including 237 patients) describing a pretty good sensitivity (80%) but a low specificity (46%). Better results were reported by Elbaum et al. (2001) [47] in a series of 246 patients; in this case, the authors used the MelaFind^® ^(Electro-Optical Sciences Inc., Irvington, New York, USA), a multispectral digital dermoscope with a function similar to the others spectrophotometers, which was described to achieve a sensitivity of 100% and a specificity of 85%. Analogously, high values of sensitivity and specificity were presented by two others studies (Moncrieff et al., 2002 [38]; Govindan et al., 2007 [49]); however, Moncrieff and colleagues concluded that the SIAscope did not add accuracy in melanoma diagnosis in comparison with the dermatologist's long experience.

**Table 9:** Summary of the most important studies on sensitivity and specificity of SPT

Reference	N. Patients	Sensitivity	Specificity
Tomatis S et al., 1998 [44]	**44**	**89%**	**88%**
Farina B et al., 2000 [45]	**237**	**80%**	**46%**
Wallace VP et al., 2000 [46]	**47**	**84%**	**91%**
Elbaum M et al., 2001 [47]	**246**	**100%**	**85%**
Moncrieff M et al., 2002 [38]	**311**	**83%**	**80%**
Haniffa MA et al., 2007 [48]	**860**	**87%**	**91%**
Govindan K et al., 2007 [49]	**886**	**94%**	**80%**
Glud M et al., 2009 [50]	**65**	**100%**	**59%**
The present study	**54**	**66%**	**76%**

Consistently with this latter indication, Haniffa et al. (2007) [48] reported high values of sensitivity (87%) and specificity (91%) for SPT among 860 lesions examined using the SIAscope spectrophotometer; when the same lesions were however evaluated by a dermatologist with a dermatoscope and at least 3 years of experience, sensitivity and specificity resulted to be even higher (94% and 91%, respectively). Since diagnostic accuracy of dermoscopy and SPT was similar, again authors suggested that a spectrophotometer does not improve the diagnostic ability of experienced dermatologists [48]. Finally, the recent study by Glud et al. (2009) [50] revealed a 100% sensitivity but a 59% specificity for SPT (as in Farina et al., 2000 [45]), further indicating that such an approach may overestimate the proportion of possible malignant lesions (high amount of false positives).

Although the SPT is still considered as a valuable diagnostic tool, the low specificity of such a methodology (probably, due to the interference of seborrhoeic keratoses, which are not always recognized as benign lesions) represents the main hamper for the introduction of such a diagnostic tool into the clinical practice. No seborrhoeic keratosis was instead present in our series; therefore, we may speculate that the difference in diagnostic accuracy between ELM and SPT was due to additional factors in our study. Probably, the automated software of the SPT might use some parameters that do not discriminate the features of melanoma or overestimate the role of others skin components (blood, melanin, and collagen in the infrared band) as markers of malignancy. Finally, the agreement between the two screening methods was unsatisfactory (68.5%), and the high number of false positives in group 3 and 4 makes the ELM evaluation as the best technique to help clinicians in early diagnosis of cutaneous melanoma. Consistently, SPT should be utilized in clinical trials only.

## Conclusions

The spectrophotometry (SPT) allows to accurately define the skin microarchitecture features. However, the low accuracy of SPT for either thin melanocytic lesions (particularly, those with regression) or palpable lesions (such as papillomatous and dermal naevi) clearly indicates that dermoscopy remains the best diagnostic tool for the preoperative diagnosis of pigmented skin lesions.

## Competing interests

PAA participated to advisory board of the Bristol Myers Squibb and GSK; he received honoraria from Schering Plough. The remaining authors declare that they have no competing interests.

## Authors' contributions

PAA: Conception and design, manuscript writing. MP: Data analysis and interpretation, provision of study material/patients. FA: Data analysis and interpretation, provision of study material/patients. IDM: Collection and assembly of data. CC: Provision of patients, collection and assembly of data. AD: Provision of patients. ES: Provision of patients. SM: Provision of patients. MDG: Provision of patients. AV: Provision of patients. GP: Manuscript writing. NM: Conception and design, final approval of manuscript. All authors were involved in manuscript writing and provided final approval of the manuscript.

## Pre-publication history

The pre-publication history for this paper can be accessed here:

http://www.biomedcentral.com/1471-5945/10/5/prepub

## References

[B1] Kim-Schulze S Taback B Kaufman HL Cytokine therapy for cancer Surg Oncol Clin N Am 2007 16 793 818 10.1016/j.soc.2007.07.011 18022545

[B2] de Vries E Coebergh JW Cutaneous malignant melanoma in Europe Eur J Cancer 2004 40 2355 66 10.1016/j.ejca.2004.06.003 15519506

[B3] Ferlay J Bray F Pisani P Parkin DM GLOBOCAN 2002: Cancer Incidence, Mortality and Prevalence Worldwide IARC CancerBase No. 5 2004 IARCPress, Lyon version 2.0

[B4] National Cancer Institute Surveillance Epidemiology and End Results (SEER) Fact sheet http://seer.cancer.gov/statfacts/html/melan.html [cited 2009 7 July];

[B5] Parkin DM Bray F Ferlay J Pisani P Global cancer statistics, 2002 CA Cancer J Clin 2005 55 74 108 10.3322/canjclin.55.2.74 15761078

[B6] Eggermont AM Kirkwood JM Re-evaluating the role of dacarbazine in metastatic melanoma: what have we learned in 30 years? Eur J Cancer 2004 40 1825 36 10.1016/j.ejca.2004.04.030 15288283

[B7] Balch CM Gershenwald JE Soong SJ Thompson JF Atkins MB Byrd DR Buzaid AC Cochran AJ Coit DG Ding S Eggermont AM Flaherty KT Gimotty PA Kirkwood JM McMasters KM Mihm MC Jr Morton DL Ross MI Sober AJ Sondak VK Final version of 2009 AJCC melanoma staging and classification J Clin Oncol 2009 27 6199 206 10.1200/JCO.2009.23.4799 19917835 PMC2793035

[B8] Carli P De Giorgi V Soyer HP Stante M Mannone F Giannotti B Dermatoscopy in the diagnosis of pigmented skin lesions: a new semiology for the dermatologist J Eur Acad Dermatol Venereol 2000 14 353 69 10.1046/j.1468-3083.2000.00122.x 11305377

[B9] Marghoob AA Swindle LD Moricz CZ Sanchez N Slue S Halpern A Kopf A Instruments and new technologies for the in vivo diagnosis of melanoma J Am Acad Dermatol 2003 49 777 97 10.1016/S0190-9622(03)02470-8 14576657

[B10] Steiner A Pehamberger H Wolff K In vivo epiluminescence microscopy of pigmented skin lesions. II. Diagnosis of small pigmented skin lesions and early detection of malignant melanoma J Am Acad Dermatol 1987 17 584 9 1 10.1016/S0190-9622(87)70240-0 3668003

[B11] Cascinelli N Ferrario M Bufalino R Zurrida S Galimberti V Mascheroni L Bartoli C Clemente C Results obtained by using a computerized image analysis system designed as an aid to diagnosis of cutaneous melanoma Melanoma Res 1992 2 163 70 10.1097/00008390-199209000-00004 1450670

[B12] Nachbar F Stolz W Merkle T Cognetta AB Vogt T Landthaler M Bilek P Braun-Falco O Plewig G The ABCD rule of dermatoscopy. High prospective value in the diagnosis of doubtful melanocytic skin lesions J Am Acad Dermatol 1994 30 55 1 9 8157780 10.1016/s0190-9622(94)70061-3

[B13] Cristofolini M Zumiani G Bauer P Cristofolini P Boi S Micciolo R Dermatoscopy: usefulness in the differential diagnosis of cutaneous pigmentary lesions Melanoma Res 1994 4 391 4 10.1097/00008390-199412000-00008 7703719

[B14] Binder M Steiner A Schwarz M Knollmayer S Wolff K Pehamberger H Application of an artificial neural network in epiluminescence microscopy pattern analysis of pigmented skin lesions: a pilot study Br J Dermatol 1994 130 460 5 10.1111/j.1365-2133.1994.tb03378.x 8186110

[B15] Binder M Schwarz M Winkler A Steiner A Kaider A Wolff K Pehamberger H Epiluminescence microscopy. A useful tool for the diagnosis of pigmented skin lesions for formally trained dermatologists Arch Dermatol 1995 131 286 9 1 10.1001/archderm.131.3.286 7887657

[B16] Soyer HP Smolle J Leitinger G Rieger E Kerl H Diagnostic reliability of dermoscopic criteria for detecting malignant melanoma Dermatology 1995 190 25 30 10.1159/000246629 7894091

[B17] Argenziano G Fabbrocini G Carli P De Giorgi V Sammarco E Delfino M Epiluminescence microscopy for the diagnosis of doubtful melanocytic skin lesions. Comparison of the ABCD rule of dermatoscopy and a new 7-point checklist based on pattern analysis Arch Dermatol 1998 134 1563 70 10.1001/archderm.134.12.1563 9875194

[B18] Ascierto PA Satriano RA Palmieri G Parasole R Bosco L Castello G Epiluminescence microscopy as useful approach in the early diagnosis of cutaneous malignant melanoma Melanoma Res 1998 8 529 37 10.1097/00008390-199812000-00008 9918415

[B19] Binder M Kittler H Seeber A Steiner A Pehamberger H Wolff K Epiluminescence microscopybased classification of pigmented skin lesions using computerized image analysis and an artificial neural network Melanoma Res 1998 8 261 6 10.1097/00008390-199806000-00009 9664148

[B20] Seidenari S Pellacani G Pepe P Digital videomicroscopy improves diagnostic accuracy for melanoma J Am Acad Dermatol 1998 39 175 81 10.1016/S0190-9622(98)70070-2 9704824

[B21] Kittler H Seltenheim M Dawid M Pehamberger H Wolff K Binder M Morphologic changes of pigmented skin lesions: a useful extension of the ABCD rule for dermatoscopy J Am Acad Dermatol 1999 40 558 62 10.1016/S0190-9622(99)70437-8 10188673

[B22] Dal Pozzo V Benelli C Roscetti E The seven features for melanoma: a new dermoscopic algorithm for the diagnosis of malignant melanoma Eur J Dermatol 1999 9 303 8 10356410

[B23] Benelli C Roscetti E Pozzo VD Gasparini G Cavicchini S The dermoscopic versus the clinical diagnosis of melanoma Eur J Dermatol 1999 9 470 6 10491506

[B24] Lorentzen H Weismann K Petersen CS Larsen FG Secher L Skødt V Clinical and dermatoscopic diagnosis of malignant melanoma. Assessed by expert and non-expert groups Acta Derm Venereol (Stockh) 1999 79 301 4 10.1080/000155599750010715 10429989

[B25] Bauer P Cristofolini P Boi S Burroni M Dell'Eva G Micciolo R Cristofolini M Digital epiluminescence microscopy: usefulness in the differential diagnosis of cutaneous pigmentary lesions. A statistical comparison between visual and computer inspection Melanoma Res 2000 10 345 9 10.1097/00008390-200008000-00005 10985668

[B26] Ascierto PA Palmieri G Celentano E Parasole R Caracò C Daponte A Chiofalo MG Melucci MT Mozzillo N Satriano RA Castello G Sensitivity and specificity of epiluminescence microscopy: evaluation on a sample of 2731 excised cutaneous pigmented lesions. The Melanoma Cooperative Study Br J Dermatol 2000 142 893 8 10.1046/j.1365-2133.2000.03468.x 10809845

[B27] Piccolo D Ferrari A Peris K Diadone R Ruggeri B Chimenti S Dermoscopic diagnosis by a trained clinician vs. a clinician with minimal dermoscopy training vs. computer-aided diagnosis of 341 pigmented skin lesions: a comparative study Br J Dermatol 2002 147 48 1 6 10.1046/j.1365-2133.2002.04978.x 12207587

[B28] Carli P De Giorgi V Argenziano G Palli D Giannotti B Pre-operative diagnosis of pigmented skin lesions: in vivo dermoscopy performs better than dermoscopy on photographic images J Eur Acad Dermatol Venereol 2002 16 339 46 10.1046/j.1468-3083.2002.00470.x 12224689

[B29] Rubegni P Burroni M Cevenini G Perotti R Dell'Eva G Barbini P Fimiani M Andreassi L Digital dermoscopy analysis and artificial neural network for the differentiation of clinically atypical pigmented skin lesions: a retrospective study J Invest Dermatol 2002 119 471 4 10.1046/j.1523-1747.2002.01835.x 12190872

[B30] Blum A Rassner G Garbe C Modified ABC-point list of dermoscopy: a simplified and highly accurate dermoscopic algorithm for the diagnosis of cutaneous melanocytic lesions J Am Acad Dermatol 2003 48 672 8 10.1067/mjd.2003.282 12734495

[B31] Soyer HP Argenziano G Zalaudek I Corona R Sera F Talamini R Barbato F Baroni A Cicale L Di Stefani A Farro P Rossiello L Ruocco E Chimenti S Three-point checklist of dermoscopy. A new screening method for early detection of melanoma Dermatology 2004 208 27 31 10.1159/000075042 14730233

[B32] Barzegari M Ghaninezhad H Mansoori P Taheri A Naraghi ZS Asgari M Computeraided dermoscopy for diagnosis of melanoma BMC Dermatol 2005 5 8 10.1186/1471-5945-5-8 16000171 PMC1177938

[B33] Menzies SW Bischof L Talbot H Gutenev A Avramidis M Wong L Lo SK Mackellar G Skladnev V McCarthy W Kelly J Cranney B Lye P Rabinovitz H Oliviero M Blum A Varol A De'Ambrosis B McCleod R Koga H Grin C Braun R Johr R The performance of SolarScan: an automated dermoscopy image analysis instrument for the diagnosis of primary melanoma Arch Dermatol 2005 141 1388 96 [Erratum in Arch *Dermatol *2006;**142**:558] 10.1001/archderm.141.11.1388 16301386

[B34] Carli P Mannone F De Giorgi V Nardini P Chiarugi A Giannotti B The problem of false-positive diagnosis in melanoma screening: the impact of dermoscopy Melanoma Res 2003 13 179 182 10.1097/00008390-200304000-00011 12690302

[B35] Kittler H Pehamberger H Wolff K Binder M Diagnostic accuracy of dermoscopy Lancet Oncol 2002 3 159 65 10.1016/S1470-2045(02)00679-4 11902502

[B36] Vestergaard ME Macaskill P Holt PE Menzies SW Dermoscopy compared with naked eye examination for the diagnosis of primary melanoma: a meta-analysis of studies performed in a clinical setting Br J Dermatol 2008 159 669 76 18616769 10.1111/j.1365-2133.2008.08713.x

[B37] Rajpara SM Botello AP Townend J Ormerod AD Systematic review of dermoscopy and digital dermoscopy/artificial intelligence for the diagnosis of melanoma Br J Dermatol 2009 161 591 604 10.1111/j.1365-2133.2009.09093.x 19302072

[B38] Moncrieff M Cotton SD Claridge E Hall PN Spectrophotometric intracutaneous analysis - a new technique for imaging pigmented skin lesions Br J Dermatol 2002 146 448 57 10.1046/j.1365-2133.2002.04569.x 11952545

[B39] Cotton SD Claridge E Developing a predictive model of human skin colouring Proc SPIE 1996 2708 814 25 full_text

[B40] Cotton SD Claridge E Hall PN Duncan J, Gindi G Non-invasive skin imaging Proceedings of Information Processing in Medical Imaging 1997 London: SpringerVerlag 501 6

[B41] Marshall RJ Infrared and ultraviolet photography in a study of the selective absorption of radiation by pigmented lesions of skin Med Biol Illus 1976 26 71 84 948213

[B42] Ascierto PA Palmieri G Botti G Satriano RA Stanganelli I Bono R Testori A Bosco L Daponte A Caraco C Chiofalo MG Melucci MT Calignano R Tatangelo F Cochran AJ Castello G Melanoma Cooperative Group Early diagnosis of malignant melanoma: Proposal of a working formulation for the management of cutaneous pigmented lesions from the Melanoma Cooperative Group Int J Oncol 2003 22 1209 15 12738985

[B43] Marchesini R Bono A Tomatis S Bartoli C Colombo A Lualdi M Carrara M In vivo evaluation of melanoma thickness by multispectral imaging and an artificial neural network. A retrospective study on 250 cases of cutaneous melanoma Tumori 2007 93 170 7 17557564 10.1177/030089160709300210

[B44] Tomatis S Bartoli C Bono A Cascinelli N Clemente C Marchesini R Spectrophotometric imaging of cutaneous pigmented lesions: discriminant analysis, optical properties and histological characteristics J Photochem Photobiol B 1998 42 32 9 10.1016/S1011-1344(97)00113-9 9491594

[B45] Farina B Bartoli C Bono A Colombo A Lualdi M Tragni G Marchesini. Multispectral imaging approach in the diagnosis of cutaneous melanoma: potentiality and limits Phys Med Biol 2000 45 1243 54 10.1088/0031-9155/45/5/312 10843103

[B46] Wallace VP Bamber JC Crawford DC Ott RJ Mortimer PS Classification of reflectance spectra from pigmented skin lesions, a comparison of multivariate discriminant analysis and artificial neural networks Phys Med Biol 2000 45 2859 71 10.1088/0031-9155/45/10/309 11049176

[B47] Elbaum M Kopf AW Rabinovitz HS Langley RG Kamino H Mihm MC Jr Sober AJ Peck GL Bogdan A Gutkowicz-Krusin D Greenebaum M Keem S Oliviero M Wang S Automatic differentiation of melanoma from melanocytic nevi with multispectral digital dermoscopy: a feasibility study J Am Acad Dermatol 2001 44 207 18 10.1067/mjd.2001.110395 11174377

[B48] Haniffa MA Lloyd JJ Lawrence CM The use of a spectrophotometric intracutaneous analysis device in the real-time diagnosis of melanoma in the setting of a melanoma screening clinic Br J Dermatol 2007 156 1350 2 10.1111/j.1365-2133.2007.07932.x 17535234

[B49] Govindan K Smith J Knowles L Harvey A Townsend P Kenealy J Assessment of nurseled screening of pigmented lesions using SIAscope J Plast Reconstr Aesthet Surg 2007 60 639 45 10.1016/j.bjps.2006.10.003 17485052

[B50] Glud M Gniadecki R Drzewiecki KT Spectrophotometric intracutaneous analysis versus dermoscopy for the diagnosis of pigmented skin lesions: prospective, double-blind study in a secondary reference centre Melanoma Res 2009 19 176 9 10.1097/CMR.0b013e328322fe5f 19319002

